# Ethnobotanical appraisal and medicinal use of plants in Patriata, New Murree, evidence from Pakistan

**DOI:** 10.1186/1746-4269-9-13

**Published:** 2013-02-27

**Authors:** Ejaz Ahmed, Muhammad Arshad, Abdul Saboor, Rahmatullah Qureshi, Ghazala Mustafa, Shumaila Sadiq, Sunbal Khalil Chaudhari

**Affiliations:** 1 Department of Botany, PMAS-Arid Agriculture University Rawalpindi, Rawalpindi, Pakistan; 2 Department of Economics, PMAS-Arid Agriculture University Rawalpindi, Rawalpindi, Pakistan

**Keywords:** Ethnobotany, Medicinal use of plants, Probabilities, Logit expression, Patriata, Murree, Pakistan

## Abstract

**Background:**

This paper reflects the empirical findings of an ethnobotanical survey which was undertaken in Patriata (New Murree) of district Rawalpindi in Pakistan. The aims and objectives of the study were to document indigenous knowledge of plants particularly of medicinal, veterinary, fruit, vegetable, fodder, fuel etc.

**Methods:**

For this purpose, the whole area was surveyed for documenting folk knowledge using a semi-structured questionnaire. A total of 93 plants species belonging to 80 genera and 56 families were found in a variety of uses by the local people for the accomplishment of their basic needs. The study further employs binary logit regression model of medicinal uses of these plants so as to identify the probability of occurrence of medicinal use of woody or non-woody plants keeping other plant characteristics in view.

**Results:**

Ethnobotanical data shows that most plants are used for medicinal and fodder purposes (27.93% each), followed by fuel (16.90%), fruit (6.55%), vegetable (5.52%) and ethno-veterinary (3.79%). There is also an established association of medicinal use of plants to the fruits use. Non-woody plants have high tendency towards medicinal use of the plants as compared to woody plants. Annual plants are less likely to be directly associated with medicinal use of plants in the surveyed vegetation. Underground plant parts are more likely to be used for medicinal purposes as revealed from the Logit expressions.

**Conclusions:**

The study revealed that most of the plants are used for medicinal and fodder purposes. The results of Logit Model showed that the probabilities of plant species for their medicinal use are associated to the woody or non-woody, aerial or underground, perennial or annual characteristics of plants. One should be careful in completely generalizing the results as the survey findings are sensitive to the plant species and the vegetation under consideration. But it can be specified that there exists either some positive or negative association of medicinal use of plants to the various characteristics of plant species.

## Background

Ethnobotany accounts for the study of relationship between people and plants for their use as medicines, food, shelter, clothing, fuel, fodder and other household purposes [1]. It deals with the interaction of indigenous plants and the local inhabitants of the area. The aim of ethnobotanists is to explore how these plants are used as food, clothing, shelter, fodder, fuel, furniture and how medicinal use of such plants is associated to other characteristics of the plant species. It is a multidisciplinary science that studies “the relationship between a given society and its environment and in particular the plant world”. They understand and collect the knowledge of valuable plants by the use of anthropological methods [2].

Humans are mainly dependant on plants for medicine and therapeutics and still 70 percent of the world population depends on medicinal plants for their primary healthcare needs [3]. Preservation and enhancement of indigenous plant knowledge is actually rescuing a global heritage [4]. Ethnobotanical studies in various areas of Pakistan have been carried out [5-9].

Since the advancement in the field of ethnobotany, importance of traditional ethnobotanical knowledge in the traditions and culture of rural populations have fully been realized and documented in most parts of the world. But in developing countries where populations are more dependants upon traditional ethnobotanical knowledge, the understanding of this fact needs to be matured.

The present study was aimed to explore the traditional utilization of plants of Patriata, New Murree located in district Rawalpindi. The study area is part of country’s richest biodiversity centre and a source of ethnobotanical knowledge. Most of the population of the area is rural with low literacy rate and they also lack modern health facilities, hence they are more dependant upon natural resources especially plants for their healthcare and to compensate their low income as well. Topographically the area mainly comprises hills and slopes and therefore very little accessed for research studies. The present study would prove very fruitful in depicting the traditional affiliation and dependence of rural people with plant resources of the area. The study further explored the probabilistic association of medicinal use of such plants with other peculiar characteristics of plants including some other domestic uses.

## Method

### Research area, climate and vegetation

Patriata is a famous hill station located at about 65Km North East of Islamabad. It is located at 33° 51’ N latitude and 73° 28’ E longitudes and it is present at an altitude of 2100-2743 Meters above sea level. Patriata is the highest place in the area and the hill top stands 2743 Meters above from sea level. Most of the area comprises mountain slopes with soil derived from weathering of bedrock resulting in mixed residuum and colluvium. Due to its location at high altitude, the climate usually remains pleasant from April to September, while it becomes extreme cold type during October to March. Snowfall usually happens from mid December to February and the temperature may drop up to -10°C [10].

The area falls under Sub-tropical and Moist temperate forests in which Chir Pine (*Pinus roxburghii*) and blue pine (*P*. *wallichiana*) are the most dominant tree species in the area. Due to cool and humid conditions for most of the year, the vegetation in the area comprises a wide variety of trees, herbs, shrubs and climbers. Ground cover comprises a wide variety of angiosperms along with ferns and mosses.

### Field work and collection of data

The ethnobotanical knowledge was documented through a semi-structured questionnaire. During field visits, interviews were conducted from 37 local people especially older people and rural herbalists (Hakeems) who were familiar with traditional uses of plants particularly for medicinal, veterinary, fruit, vegetable, fodder, fuel and others. The queries were repeatedly made to increase the reliability of the data. Identification of plant samples was done by using the available literature [11-14]. Plant specimens were collected, pressed, dried and identified in the Herbarium of, Quaid-i-Azam University Islamabad. After proper identification, these plant specimens were deposited in the Herbarium, Pir Mehr Ali Shah Arid Agriculture University Rawalpindi as voucher specimen for future references.

### Logit model

Logit expression is helpful in understanding the in-depth probabilistic relationships among various variables. In the present case, medicinal use is attributed to various characteristics and traits of the plants. By analyzing data through logit model we become able to empirically estimate the probability of medicinal use of plants due to woody and non-woody, aerial and underground, perennial and annual nature of plants in addition to the use of plant as fodder or as fruit.

There is wide use of Logit Model in Plant and Animal sciences particularly to verify the probability of occurrence of an event (for instance, the probability of use of woody or non-woody plants in medicinal use). By using this model we become confident to relate the characteristics of plant species in a particular ecosystem. This is the inherent beauty of the model that it takes into account the exogenous variables like overall vegetation in a particular ecosystem. In order to incorporate taxonomies of different plant species in the same model, a huge set of data and associated information is required which is beyond the scope of this study. At least, we authenticate our findings by employing Logit specifications which otherwise remain a perception and indigenous observation. The advantage of Logit results are that the findings can be applied and specified for having a broad picture in other ecosystems as well. Moreover, the typical probabilistic relationships further help in pursuing research on important variables across regions of the similar flora.

The study employs binary logit regression model of medicinal use of 93 plants species of Patriata region because we consider the case where response Medicinal Use (MU) is binary that will take the value of 1 if yes is associated with the plant; zero (0) is taken if there is no medicinal use of plant. The logit model for medicinal use of plant thus took the form

$$\text{Logit}\left(L_{i}\right)=\beta_{0}+\sum_{j=1}^{k}\beta_{i}X_{\text{ij}}+\mu_{i}$$

Where dependent variable Logit (L_i_) is log of odd ratios and Xi is the vector of all explanatory variables used in the regression analysis i.e. Woody and Non-Woody (WDN) Plants (by taking 1 for Woody Plants and 0 for non-woody Plants), Perennial and Annual (PAN) plants (by taking 1 for Perennial Plants and 0 for Annual Plants), Aerial and Undergrounds (AUG) plant parts (by taking 1 for Aerial Plant parts and 0 for Underground Plant parts), use of plant as Fruit (by taking 1 if there is use of plant as fruit otherwise 0) and fodder (by taking 1 if there is use of plant as fodder otherwise 0) while Li is constructed for Medicinal Use (MU) of plant.

Dependent variable in logit model is logit rather than a mean so coefficients are in logit. βi represents the change in the logit of the probability associated with a unit change in the respective predictor holding all other predictors constant [15]. These types of interpretations are unfamiliar so an appropriate way is to interpret the marginal effects in the logit model. We constructed Marginal Effects for our analysis. Hence, to interpret parameter estimates through marginal effect, the slope coefficients of the logit model were transformed to yield estimates of the marginal effects i.e. the change in the predicted probability associated with the change in the covariates [16,17].

## Results and discussion

### Ethnobotanical importance

A total of 93 plant species belonging to 80 genera and 56 families are reported in the present communication being used by the natives for multi-purpose. The detailed inventory is provided in Table 1, which includes botanical names, followed by local name, family and ethnobotanical uses.

**Table 1:** Ethnobotanical uses of plants of Patriata, New Murree

**S. no**	**Plants names/voucher number**	**Local name**	**Family**	**Ethnobotanical uses**
01	***Acacia catechu *(L.f.) Willd/ej-03**	Kikar	Mimosaceae	Bark along with leaves of *Olea ferruginea* were used for tea making by the people in early days used for treating skin diseases and as cooling agent. Wood is used in roof thatching, fuel wood, coal formation and fencing.
02	***Acacia modesta *L./ej-04**	Phulai	Mimosaceae	Branches are used as tooth stick (*Miswak*) for teeth cleansing and tooth decay. The gum is used as tonic and given in general weakness. Wood is used for agricultural implements e.g. Hull, fuel, branches used for fencing fields and leaves are browsed by goats.
03	***Achyranthis aspera *L./ej-06**	Puth Kanda, Chooroon	Amaranthaceae	Roots and leaves are boiled in water to make decoction, which is given in toothache and digestive problems. Leaf paste is applied externally on insect bite. The powder of roots is used in bloody diarrhoea.
04	***Adiantum capilus*-*veneris *L./ej-07**	Hansraal	Adiantaceae	Decoction of leaves is prescribed in cold, cough, flue and asthma.
05	***Aesculus indica *(Wall ex Camb.) Hook. f./ej-09**	Ban khor	Hippocastinaceae	Oil extracted from fresh fruits is applied externally on wounds. The powder of seeds is taken orally against acidity and digestive problems for both humans and animals. Leaves are used as fodder and dried branches are used for fuel source.
06	***Ajuga bracteosa *Wall. Ex Benth./ej-11**	Guchi	Lamiaceae	The leaf paste is used orally against constipation.
07	***Aloe vera *(L.) Burm f./ej-12**	Kanwar gandal	Aloaceae	The salt is dusted on pulp and kept overnight under moonlight which is given early morning to treat tumours in the digestive tract. The same is given as an appetizer and tonic. As an appetizer and treatment of leprosy, the same is given to cattle.
08	***Alternanthera pungens *Kunth./ej-13**	Lundri	Amaranthaceae	The powder of plant is used in jaundice. Whole plant is used for fodder.
09	***Amaranthis viridis *L./ej-14**	Ganiar	Amaranthaceae	Fresh green leaves are used as potherb and given to treat constipation. Aerial parts are used as fodder and leaves are given to young animals to induce puberty.
10	***Artimesia scoparia *Waldst & Kit./ej-22**	Chahoo	Asteraceae	Juice of fresh leaves mixed with brown sugar and given orally in malarial fever, skin disease like scabies, pimples and supposed to possess cooling effects. The plant is used as fodder.
11	***Asparagus adscendense *Roxb./ej-24**	Sumbloo, Sufaid Musli	Asparagaceae	The extract of tuberous roots is used in diarrhoea and dysentery. Tubers are also used for animal problems. Leaves are browsed by goats.
12	***Bauhinia variegata *L./ej-30**	Kachnar, Kuliarh	Caesalpinaceae	Buds locally called “*Kalian*” are used as vegetable given in digestive problems. The leaves are used as fodder and branches as fuel.
13	***Berberis lycium *Royle./ej-31**	Sumbal	Berberidaceae	The paste of root bark is externally applied on wounds. Powdered bark is mixed in water and the paste is applied on bone fracture. Crushed bark is soaked in water and the resultant extract is taken early morning to treat diabetes, scabies, boils and pimples. The extract possesses cooling effect and seldom used in winter season. Fruits are edible. Leaves are used as fodder and dried branches for fuel.
14	***Bergenia ciliata *(Haw.) Sternb./ej-32**	Bhat-Phay	Saxifragaceae	The powder of roots is given along with *Deesi* ghee in diabetes and skin diseases. The same is believed in reducing blood cholesterol.
15	***Bergenia stracheyi *(Hook f. & Thoms.) Engle./ej-33**	Bhat-Phay	Saxifragaceae	The powder of roots is dusted on wounds. The powder of roots is given with milk orally early morning for digestive ulcers. Aerial parts of the plant are grazed by animals.
16	***Bombax ceiba *L./ej-36**	Sanbal	Bombacaceae	Large pieces of bark are removed and tied over the wounds to heal. The wood is used for fuel source. Cottony fibres attached with seeds used in stuffing pillows.
17	***Broussonetia papyrifera *(L.) L’Herit ex Vent./ej-37**	Jangli Toot	Moraceae	This plant is planted in sliding areas because of its rapid vegetative propagation. Leaves are used as fodder and other parts for fuel wood.
18	***Calotropis procera *(Willd.) R. Br./ej-39**	Aak	Asclepiadaceae	The leaves are warmed and tied over the wounds and used as poultice for their quick healing. The latex from stem and leaves is applied upon teeth to get rid of the worms. Extreme care must be taken because latex is extremely poisonous. Latex is applied externally on skin diseases.
19	***Carrisa opaca *Stapf. Ex. Haines/ej-42**	Garanda	Apocynaceae	Decoction of fresh leaves is used against hepatitis and jaundice. Fruits are edible and used as digestive stimulant. Fruits are edible and also sold by poor people for generating their income. Dried branches are used for fuel and the leaves are browsed by goats.
20	***Cassia fistula *L./ej-43**	Amaltas	Caesalpinaceae	The pulp of fruits is used against constipation. Leaves are used as fodder and dried branches are used for fuel. The fruits are collected by local herb sellers called “Pensaries” and Hakeems and used in various herbal medicines. Pulp of fruits is also used to relieve constipation in cattle.
21	***Cedrus deodara *(Royle. ex Lamb.) Loud./ej-44**	Diarr, Deodar	Pinaceae	Small pieces of stem are boiled in water to get oily extract called “Lou” which is used as aphrodisiac. The wood of plant is used for making doors, windows and cupboards. It is considered very unique for interior wood-work due to its characteristic smell, insect resistance and is also very expensive.
22	***Chinopodium album *L./ej-48**	Bathu, Karhan saag	Chinopodiaceae	Fresh green leaves are used as spinach. It is used as a digestive stimulant. Leaves are used as fodder.
23	***Cissampelos pareira *L./ej-52**	Ghorhi Sumbi, Pla jarrhi	Menispermaceae	Fresh leaves are crushed and the extract mixed with sugar is used against diarrhoea and dysentery. Leaves are used as fodder.
24	***Convolvulus arvensis *L./ej-58**	Lily	Convolvulaceae	Leaves are used as spinach to get rid of intestinal worms. The plant is having purgative effect and is also used against constipation.
25	***Cotinus coggyra *Scop./ej-63**	Bhann, Phann.	Anacardiaceae	The leaves are used for fodder purpose. Dried branches are used for fuel wood. Its young branches are used to make baskets. It branches are twisted to be used as ropes.
26	***Dabregeasia salicifolia *(D. Don) Rendle/ej-68**	Sindwaar	Urticaceae	The fruits are grinded and are used against bloody diarrhoea. Leaves and branches are used as fodder.
27	***Dalbergia sissoo *Roxb./ej-71**	Shishum, Taali	Papilionaceae	Its plant is excellent source of furniture wood. The wood is highly durable and insect resistant. Wood is also used for fuel.
28	***Diosypros lotus *L./ej-74**	Amlok	Ebenaceae	The fruits are edible and are used against stomach problems and dyspepsia. The leaves are used as fodder and dried branches for fuel.
29	***Dodonea viscosa *(L.) Jacq./ej-75**	Sanatha	Sapindaceae	The leaves are boiled in water and steam is inhaled to get relief from respiratory problems like cold, cough and asthma. Dried branches are used for fuel for producing heat without smoke.
30	***Dryopteris ramosa *(C. Hope) C. Chr./ej-76**	Pakha, Pakhi	Dryopteridaceae	Collection of Young leaves is made in spring season and used as vegetable. It is effective against gastric ulcer and constipation. Leaves are used as fodder.
31	***Duchesnia indica *(Andr.) Focke/ej-77**		Rosaceae	Fruits paste is used against bloody diarrhoea. Whole plant is also used as fodder.
32	***Echinopes echinatus *Roxb./ej-78**	Hand, Barhong	Asteraceae	Leaves are boiled and the decoction is taken orally against swelling in the body. The leaves are also used as spinach. Aerial parts are used as fodder for camels.
33	***Emblica officinalis *Gaertn. Fruct./ej-79**	Amla	Euphorbiaceae	Fruits are boiled, the pulp is dried and stored and later on used for making curry called “Chitt”. It is also a strong digestive stimulant and also has a cooling effect. Extract of the dried fruits alone or mixed with yoghurt is also used against jaundice. Leaves are browsed by goats. Dried wood is used for fuel. Fruits are sold in the market.
34	***Eruca sativa *L./ej-82**	Tara Meera	Brassicaceae	The leaves and young branches are used as spinach and are believed to be effective against skin diseases, constipation and digestive ulcers. Whole plant is used for fodder.
35	***Ficus glomerata *Roxb./ej-87**	Phagwarh	Moraceae	Young leaves are tasted, if not bitter are collected as “Phagwalla” and cooked in “Lassi” (remains of milk after extracting butter) and is especially effective in treating intestinal problems. Fruits are edible and are effective against constipation. Fruits called “Phagwara” are edible. Branches are used as fuel.
36	***Ficus virgata *Reinwardt. ex Blume/ej-90**	Tussi	Moraceae	Fruits are edible and are effective against digestive problems especially constipation. The young leaves called “barh kandlaan” are cooked in “Lassi” and are effective against digestive problems and have cooling effect. Dried branches are used for fuel wood.
37	***Flacourtia indica *(Burm.f.) Merrill/ej-91**	Kokoh	Flacourtiaceae	The fruits are edible and are also used against diabetes. Leaves are used as fodder and dried branches are used for fuel wood.
38	***Geranium rotundifolium *L./ej-95**	Ratan-jot	Geraniaceae	The roots are dried and grinded, sugar and milk are added in it, and it is used for pain in joints and also as antispasmodic. Its roots are grinded and along with brown sugar used against blockage of urine and also believed to be having cooling effect.
39	***Grewia optiva *Drum. Ex. Burret./ej-98**	Tamman, Dhamman	Tiliaceae	Leaves are given to cattle especially during delivery for quick discharge of afterbirth. It is also given to young animals to induce puberty. Branches are soaked in water and the detached bark is used for making ropes. Leaves are used as fodder.
40	***Hedera nepalensis *K. Koch./ej-100**	Baleri, Albhambar	Araliaceae	The dried branches and leaves are grinded and the powder is used early in the morning with water against diabetes.
41	***Ipomoea purpurea *(L.) Roth./ej-106**	Aerh	Convolvulaceae	Leaves are grinded and the extract is used for washing hairs to get rid of lices. Whole plant is used as fodder.
42	***Jasminum officinale *L./ej-108**	Chambeli	Oleaceae	The aqueous extract of leaves is externally applied on skin having scabies or any allergic problem. The plant is also planted as ornamental on graves.
43	***Juglans regia *L./ej-109**	Akhore, Akhrot	Juglandiaceae	Seeds are edible and are also effective for cardiac patients and as tonic. Pericarp of fruit, fresh leaves and bark (locally called Dandaasa) is used for cleaning of teeth and mouth ulcers. Dried wood is light weight and is used for making furniture. Wood is also used for fuel.
44	***Justicia adhatoda *L./ej-110**	Baikkarh	Acanthaceae	Leaves are grinded and dissolved in water and this extract is taken orally early morning against diabetes, scabies, boils, pimples and other skin diseases. It is having drying effect and deesi ghee is used during its use. Dried branches are used for fuel. Green Leaves are used in producing smoky fire to drive away the insects from cattle.
45	***Lepidium sativum *L./ej-120**	Halyan	Brassicaceae	Its seeds are put in eyes, which produce mucilage which is very effective in cleaning eyes and especially used to get rid of dust from eyes. When the seeds are taken out they carry these materials out.
46	***Lonicera quinquelocularis *Hardwicke/ej-121**	Phutuk	Caprifoliaceae	Fresh leaves are crushed and the extract is poured in eyes to cure the cataract and to improve vision. Fresh leaves are used as fodder for goats.
47	***Mallotus philipensis *(Lam.) Muell./ej-123**	Kamila	Euphorbiaceae	The fruits are crushed and used orally to treat bloody diarrhoea. The leaves are used as “Koochan” to wash utensils. The leaves are used as fodder and branches for fuel.
48	***Medicago polymorpha *L./ej-127**	Maeserhi, Maina	Fabaceae	Leaves and young branches are picked and used as spinach. It is also effective against constipation and other digestive problems. Whole plant is used for fodder.
49	***Melia azedarach *L./ej-128**	Dharek	Meliaceae	The leaves are grinded and the extract is used against scabies, pustules, pimples, boils and other skin diseases. Leaves are used as fodder and dried branches for fuel. It is also planted in lawns for shade in summer.
50	***Mentha arvensis *L./ej-130**	Kala Poodina	Lamiaceae	Leaves of *Mentha arvensis*, young fruits of *Zanthoxylum alatum* are grinded with seeds of *Punica granatum*, and green chillies to make “Chatni” which is carminative and have cooling effect. It is also a digestive stimulant.
51	***Mentha longifolia *(L.) Hudson/ej-131**	Sufaid Poodina	Lamiaceae	Leaves of *Mentha longifolia*, young fruits of *Zanthoxylum alatum* are grinded with seeds of *Punica granatum*, and green chillies to make “Chatni” which is carminative and have cooling effect. It is also a digestive stimulant. Dried leaves are taken orally to stop vomiting. Its leaves are added in green tea and used for digestive problems and cholera.
52	***Morus alba *L./ej-137**	Sufaid Shehtoot	Moraceae	The fruits are edible and are used as digestive stimulant and to relieve constipation and other digestive problems. The leaves are used for fodder. Wood is used for furniture and fuel.
53	***Morus nigra *L./ej-138**	Kala Shehtoot	Moraceae	The fruits are edible and are used as digestive stimulant and to relieve constipation and other digestive problems. The leaves are used for fodder. Wood is used for furniture and fuel.
54	***Myrsine Africana *L./ej-139**	Khookhal	Myrsinaceae	Grinded fruits are used against intestinal worms. Leaves are used as fodder and branches for fuel.
55	***Nerium indicum *Mill./ej-141**	Ganeera, Kaner	Apocynaceae	The branches are used as Miswak (toothbrush) to get rid of worms, but its liquid extract in the branches and leaves is highly poisonous so extreme care should be taken not to be taken orally. The plant is used as ornamental due to its beautiful flowering.
56	***Olea ferruginea *Royle./ej-143**	Kahu	Oleaceae	The leaves along with bark pieces of *Acacia catechu* were used by people in early days to make tea. It was especially used against cough, cold, flue and skin diseases. Young leaves are chewed to avoid toothache and mouth ulcers. Young branches are used as Miswak. The wood is extremely durable and is extensively used. Its elongated logs are used as guarders in roof thatching. The straight branches are used as handles for labour’s tools. The leaves are used as fodder and dry branches are used for fuel. The wood yield more heat without smoke so its wood is especially used during extreme winter. The wood is also insect resistant.
57	***Olea glandulifera *Wall. ex G. Don./ej-144**	Barh-koh	Oleaceae	The leaves are used as fodder and dry branches are used for fuel.
58	***Otostegia limbata *(Benth.) Boiss./ej-145**	Chita jand	Lamiaceae	Leaves are boiled and the extract is taken orally against mouth ulcers and skin diseases. Young leaves are also chewed against mouth ulcers. The leaves are browsed by goats.
59	***Oxalis corniculata *L./ej-146**	Khat-mith	Oxalidaceae	The leaves of the plant are crushed and the extract is used orally against jaundice. The whole plant is used for fodder.
60	***Pinus roxburgii *Sergent/ej-151**	Chirr	Pinaceae	Juvenile apex of the stem is grinded and is used against bloody diarrhoea. Tuberculosis patients are advised to keep sitting under its shade for quick recovery. The wood of the plant is used for timber and fuel purpose. The resin obtained is used in soap industry. The seeds are edible. Dried leaves and logs are used in roof thatching. The heartwood is highly inflammable and its small pieces are used for ignition purpose at homes.
61	***Pinus wallichiana *Jackson/ej-152**	Bainrh, Biarh	Pinaceae	The wood of the plant is used as timber and fuel. It is also used for obtaining resin. Dried leaves and logs are used in roof thatching.
62	***Plantago lanceolata *L./ej-153**	Batti, Chamchi patra	Plantaginaceae	The leaves are crushed and mixed with brown sugar and used as cooling agent for stomach.
63	***Punica granatum *L./ej-159**	Darruni	Punicaceae	The seeds along with young fruits of *Zanthoxylum alatum*, leaves of *Mentha longifolia* and green chillies are used to make “Chattni” which is a digestive stimulant. Its seeds are highly carminative. Extract of seeds have cooling effect and is especially used in summer. The rind of fruits is dried, powdered and mixed with sugar is used against diarrhoea for both humans and cattle. Branches are used for fuel and also for fencing the fields. Seeds are edible which are dried for making “Anar-dana” which is used as condiment.
64	***Pyrus pashia *Buch.-Ham. ex D. Don./ej-161**	Batangi	Rosaceae	The fruit is edible and is used against diarrhoea. The dried fruit may be used after crushing. Fruit is edible and the leaves are also used as fodder. Branches are used for fuel.
65	***Quercus incana *Roxb./ej-162**	Rein, Shah-baloot	Fagaceae	Pieces of bark are boiled in water to get their decoction, which is very effective against joint pain and is also having cooling effect. Elongated logs are used as guarders in roof thatching. Branches are used for fuel.
66	***Ricinus communis *L./ej-167**	Arand, Hernoli	Euphorbiaceae	The oil extracted from seeds of the plant is called “Castor oil” which is used as purgative. The leaves are used as poultice in rheumatic joints. Branches are used for fuel.
67	***Rosa brunonii *Lindl./ej-168**	Jangli Gulab	Rosaceae	Fresh flowers are externally massaged on skin infected from scabies. Flowers petals are used to make “Gulkand” which is believed to be effective against digestive and heart problems. The plant is used as ornamental.
68	***Rubus ellipticus *Smith./ej-169**	Aakha	Rosaceae	Fruit is edible and is having cooling effect. Spiny branches are used as fence around fields. Leaves are browsed by goats.
69	***Rubus fruiticosus *L./ej-170**	Aakha	Rosaceae	Fruits are edible and have cooling effect. Spiny branches are used in fencing. Leaves are browsed by goats.
70	***Rumex dentatis *L./ej-172**	Khoe, Jangli Palak	Polygonaceae	The extract of the leaves is used as antiseptic against wounds and skin problems. Young leaves are used as vegetable. The whole plant is used for fodder.
71	***Rumex haustatus *D. Don/ej-173**	Khatimmer	Polygonaceae	Leaves are grinded and used against jaundice. Decoction of roots is also used against jaundice.
72	***Rumex nepelansis *Spreng./ej-174**	Khoe, Jangli Palak	Polygonaceae	The extract of the leaves is used as antiseptic against wounds and skin problems. Young leaves are used as vegetable.
73	***Salix babylonica *L./ej-176**	Beiss	Salicaceae	Grinded roots are used for their cooling effects. The wood of the plant is used in making furniture. Dried branches are used for fuel. It is also planted in sliding areas.
74	***Sapindus mukorrossii *Gaertn./ej-178**	Retha	Sapindaceae	The fruits are soaked in water and are used in washing hairs to make them healthy and silky. Dried branches are used for fuel.
75	***Saussuria heteromala *DC./ej-180**	Kali Zeeri	Asteraceae	Seeds are grinded and used against skin diseases especially scabies, pimples etc. The plant is used for fodder.
76	***Solanum nigrum *L./ej-187**	Mako, Kach maach	Solanaceae	Leaves and fruits are cooked and used against abdominal swellings and stomach-ache. It is also used as spinach by cardiac patients.
77	***Solanum surratense *Burm. F./ej-189**	Mokrhi, Kandiari	Solanaceae	The extract of leaves is applied on body swellings to get relief. Its seeds are burnt in “Chehlum” and the smoke is inhaled to get relief from toothach. Fruits and leaves are boiled and the decoction is mixed in water and used for taking bath against skin diseases.
78	***Solanum villosum *(L.) Moench/ej-186**	Kach-maach	Solanaceae	The leaves are cooked and used as spinach by cardiac patients.
79	***Sonchus asper *(L.) Hill/ej-190**	Duddal	Solanaceae	Decoction of leaves and roots is taken orally against fever. It is also used against pimples, diabetes, scabies and other skin problems in the form of spinach. The plant is also used as fodder.
80	***Spermadictyon suaviolens *Roxb./ej-192**	Phisanni	Rubiaceae	The leaves are used as fodder, and especially browsed by goats.
81	***Swertia chiraita*** (**Wall**.) **C**.**B**. **Clarke**/**ej**-**194**	Charaita	Gentianaceae	The leaves are grinded and the paste is dissolved in water along with some brown sugar and is used against fever especially malaria.
82	***Swertia cordata *Wall**./**ej**-**195**	Charaita	Gentianaceae	The leaves paste along with some brown sugar is used against fever especially malaria.
83	***Syzygium cuminii*** (**L**.) **Skeels**/**ej**-**197**	Jaman	Myrtaceae	Fruits are edible and are believed to be effective against cardiac problems. The dried seeds are grinded and the powder is used against diabetes. The leaves are used for fodder purpose. Wood is used for fuel. Due to light weight wood is used to make furniture.
84	***Tribulus terestris *L**./**ej**-**202**	Bhakrha	Zygophyllaceae	The whole plant is dried, powdered and is use to ease menstrual flow, relieve constipation and its high dose is used in abortion.
85	***Trichodesma indica*** (**L**.) **R**.**Br**./**ej**-**203**	Hundusi, Gao-zaban	Boraginaceae	The leaves paste is mixed with water and brown sugar and is given orally against diarrhoea and dysentery.
86	***Verbena officinale *L**./**ej**-**206**	Chooroon	Verbenaceae	Leaves paste is used against rheumatic and joint pain.
87	***Viola canescense *Wall**. **ex Roxb**./**ej**-**210**	Banafsha	Violaceae	The leaves past is mixed with brown sugar to be used against cough, cold and other respiratory problems.
88	***Vitex negundo *L**./**ej**-**211**	Marvan	Verbenaceae	Decoction of leaves is used orally in very small amounts, and externally for taking bath against skin diseases. Leaves and branches are placed in stored wheat grains and other cereals to avoid insect pests. Dried branches are also used for fuel.
89	***Woodfordia fruiticosa*** (**L**.) **S**. **Kurz**./**ej**-**213**	Dhaawi, Taavi	Vitaceae	Flowers are dried and powdered. This powder is used locally by females for abortion. These are also used in fewer amounts to ease menstrual flow. Leaves are used as fodder and Branches as fuel.
90	***Xylosma longifolium *Clos**./**ej**-**215**	Batti	Flacourtiaceae	Dried branches are used for fuel. Long and straight branches are used as support for various purposes.
91	***Zanthoxylum alatum *Roxb**./**ej**-**216**	Timber, Timmer	Rutaceae	Young fruits are grinded with seeds of *Punica granatum*, leaves of *Mentha longifolia* and green chillies to make “Chatni”. Its fruits are highly carminative and also used against stomach-ach and dyspepsia. Young branches are used as “Miswak” just like toothbrush. Leaves are browsed by goats. Spiny branches are use as fence around fields. Straight branches are used as walking sticks.
92	***Zizyphus mauritiana *Lam**./**ej**-**217**	Bair, Beri	Rhamnaceae	Fruits are edible and used as digestive stimulant. Leaves are browsed by goats. The spiny branches are used as fencing the fields.
93	***Zizyphus oxyphylla Edgew***./**ej**-**218**	Amnui	Rhamnaceae	Roots are boiled in water to get decoction which is used against scabies, pustules and diabetes. Grinded roots are also used against jaundice. Fruits are edible and leaves are browsed by goats. Spiny branches are used in fencing the fields.

The study showed that people of the area are much dependant on the native flora for acquiring their basic requirements such as fodder, medicines, fruits, vegetables, fuel, furniture, roof thatching, fencing, etc. One of the major reasons is that the whole area is rural in nature and most of the people are not very well off. Therefore, most of them keep livestock along with other source of income. The analysis of the ethnobotanical data shows that a large number of plant species are used for fodder/forage purpose (27.93 percent). The area is a rangeland blessed with high number of palatable species, so there is great potential for livestock farming.

**Figure 1:**
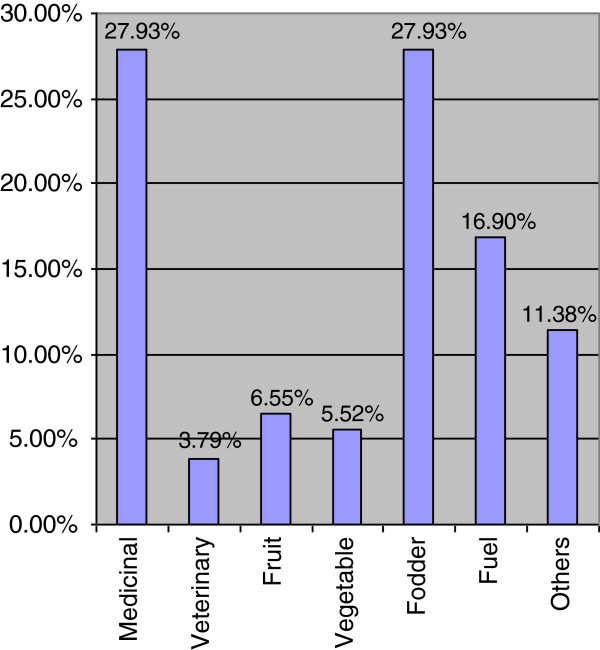
Ethnobotanical uses of flora of Patriata, New Murree.

**Figure 2:**
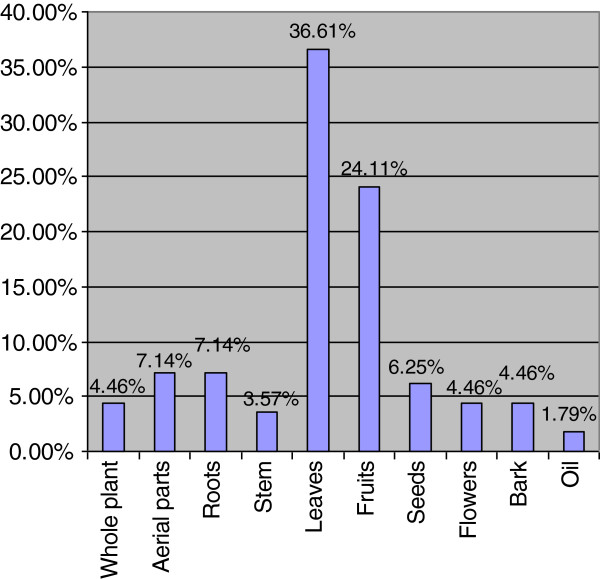
Part used for ethnomedicinal purpose of flora of Patriata, New Murree.

**Figure 3:**
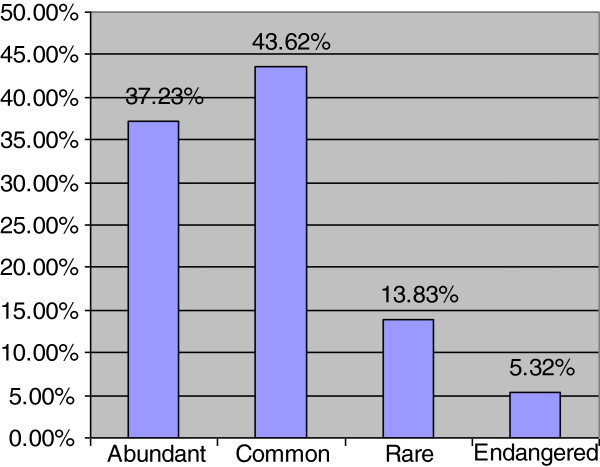
Availability of plants of Patriata, New Murree.

Ethnobotanical use categories are shown in Figure 1, which shows that almost equal proportion of species were used for medicinal as well as fodder for their domesticated animals (27.93 percent each). It was followed by fuel (16.90 percent), others (11.38 percent), wild fruit (6.55 percent), vegetable (5.52 percent) and ethno-veterinary (3.79 percent). With reference to their medicinal use (Figure 2), leaves were commonly used parts for making indigenous recipes (36.61 percent), followed by fruits (24.11 percent). Availability status of the species was also analysed and recorded in Figure 3, which shows that 37.23 percent species are abundantly present in study area, 43.62 percent species are common, 13.83 percent species are rare and 5.32 percent species are endangered in the area and need their conservation.

Due to absence of fuel source “local population” is totally dependant upon fuel wood species for their survival. They are extensively cutting forests for their fuel wood requirements without any knowledge of their extinction, so a number of species are rapidly decreasing in the study area. One way to reduce this pressure on the natural vegetation is that, people may be provided with alternate fuel sources like natural gas.

During interviews with the local people, it was noted that the ethnobotanical knowledge is becoming restricted only to the elder people, *Hakeems*, and *pensaries* (local herb sellers). Young generation is totally ignorant of this wealth. Advancement in science and technology has changed social setup; therefore young generation is leaving traditions and culture.

### Some significant findings from logit expression

The results reported in Table 2 reflect the logit estimates for medicinal use analysis for 93 plants. The table represents logit coefficients as well as odd ratios but a more comprehensive and meaningful interpretation was made through marginal effects (marginal probabilities). The effect of the variable WND shows that probability of woody plants to be used for medicinal purposes is 45.26 percentage point lower than that of non-woody plants. Thus, among the surveyed 93 species, non-woody plants have high tendency towards medicinal use of the plants as compared to woody plants (keeping the socio-economic characteristics of the communities in respective region constant). The probability of a perennial plant for medicinal use is about 95.18 percent higher than that of annual plants. So, there is likelihood of the fact that more the plant a perennial one, higher is the chance of its medicinal use in the specific region keeping all other probabilities constant. Annual plants are less likely to be directly associated with medicinal use of plants in that particular ecosystem. All such results are statistically significant.

**Table 2:** Maximum likelihood estimates for medicinal use logit regression

**Covariate**	**Coefficient**	**Odd**-**Ratio**	**Z**-**Stat**	**P**-**Value**	**Marginal effects**
Constant	21.04215	--	2.39	0.017	--
WND	−17.65597	2.15e-08	−26.66	0.000	-.452617
PAN	16.67147	1.74e+07	5.06	0.000	.9517718
AUG	−17.97821	3.96e-08	−7.07	0.000	-.0023579
FRUIT	18.09735	7.24e+07	29.13	0.000	.0199024
FODDER	−1.192138	.3035714	−0.15	0.881	-.0004085
Pseudo R^2^		0.1777			
Likelihood ratio test statistic		−29.407017			
Wald Chi-Sq (5)		848.02 (0.000)			
Replications		50			

On the other hand, probability of aerial plant parts in medicinal use is about 0.24 percent lower than underground plant parts. In other words, the aerial and underground plant parts of species of Patriata Ecosystem are almost equally eligible for the medicinal values. Similarly, the probability of fruits of plants for medicinal use is about 2 percentage point higher than non-fruit parts. The probability of fodder plants in medicinal use is 0.004 percentage point lower than non-fodder plants. This value is too low to be effective and also statistically insignificant showing that plants used as fodder are ineffective in medicinal use. All the variables are highly significant at less than 1 percent except fodder (which is statistically insignificant which might be due to the ignorance of the local community). The Wald Chi-Square test statistic is also very high with probability value less than 1 percent showing that model is highly significant. The overall significance of the model further authenticates the findings related to medicinal use of plants. All other empirical specifications including descriptive statistics have been shown in Additional file 1: Appendix-A.

One should be careful that the survey results are sensitive to the plant species and the vegetation under consideration in addition to socio-cultural characteristics of the dwellers of the region. But it can be specified that there exists either positive or negative association of medicinal use to the various characteristics of plant species. The probability of medicinal use of plants is fairly linked to the probability woody and non-woody, perennial and annual, aerial and underground characters of plants.

## Conclusion

The present study reveals that ethnobotanical knowledge is found restricted to indigenous culture, so change in traditional culture will surely result in loss of this valuable treasure. There is need of hour to document this hidden treasure to avoid its extinction and the present study is a part of this effort. Based on the present investigation, there is need to authenticate medicinal and forest products of plants on scientific lines. On the other hands, conservation status should be determined of the native flora required for conserving endangered species. It has been determined that the probabilities of plant species for their medicinal use are associated to the woody or non-woody, aerial and underground, perennial and annual characters of plants. There is also an established association of medicinal use of plants to the fruits use. Non-woody plants have high tendency towards medicinal use of the plants as compared to woody plants. Annual plants are less likely to be directly associated with medicinal use of plants in the surveyed ecosystem. A further exploration in the same fashion in other ecosystems can lead us to some solid understanding of medicinal use of plants and its probabilistic association with other features of plant species and their respective taxonomies.

## Competing interests

The authors declare no competing interests.

## Authors’ contributions

MA designed the research project, provided comments and suggestions on the draft. EA conducted the field work, analysed the data and wrote the draft of manuscript. RQ conducted the field work and provided comments on the draft. GM and SKC conducted field work and analysed the data. AS and SS have conducted statistical analysis and analysed the manuscript. All authors have read and approved the final manuscript.

## Supplementary Material

Additional file 1 **Appendix-A.** Table A-1 Descriptive Statistics. Table A-2 Coefficients in Logit Analysis. Table A-3 Odd Ratios in Logit Analysis. Table A-4 Marginal Effects in Logit Analysis. Click here for file
