# Supplementary material for: Ethnobotanical appraisal and medicinal use of plants in Patriata, New Murree, evidence from Pakistan
Source: J Ethnobiol Ethnomed. 2013 Feb 27;9:13. doi: 10.1186/1746-4269-9-13 (PMC3599915; doi:10.1186/1746-4269-9-13)
Supplement: Additional file 1 — Appendix-A. Table A-1 Descriptive Statistics. Table A-2 Coefficients in Logit Analysis. Table A-3 Odd Ratios in Logit Analysis. Table A-4 Marginal Effects in Logit Analysis. [file 1746-4269-9-13-S1.doc]

Appendix-A

Table A-1 Descriptive Statistics

Table A-2 Coefficients in Logit Analysis

Table A-3 Odd Ratios in Logit Analysis

Table A-4 Marginal Effects in Logit Analysis
